# Current Status of Biodiversity Assessment and Conservation of Wild Olive (*Olea europaea* L. subsp. *europaea* var. *sylvestris*)

**DOI:** 10.3390/plants11040480

**Published:** 2022-02-10

**Authors:** Valentina Fanelli, Isabella Mascio, Wahiba Falek, Monica Marilena Miazzi, Cinzia Montemurro

**Affiliations:** 1 Department of Soil, Plant and Food Sciences, University of Bari Aldo Moro, 70125 Bari, Italy; mascioisa@gmail.com (I.M.); cinzia.montemurro@uniba.it (C.M.); 2 Ecole Nationale Superieure de Biotechnologie, Constantine 251000, Algeria; wfalek@ymail.com; 3 Spin Off Sinagri s.r.l., University of Bari Aldo Moro, 70125 Bari, Italy; 4 Support Unit Bari, Institute for Sustainable Plant Protection, National Research Council of Italy (CNR), 70125 Bari, Italy

**Keywords:** oleaster, biodiversity, morphological evaluation, genetic analysis, molecular markers, conservation, genebanks

## Abstract

Oleaster (*Olea europaea* L. subsp. *europaea* var. *sylvestris*) is the ancestor of cultivated olive (*Olea europaea* L. subsp. *europaea* var. *europaea*) and it is spread through the whole Mediterranean Basin, showing an overlapping distribution with cultivated olive trees. Climate change and new emerging diseases are expected to severely affect the cultivations of olive in the future. Oleaster presents a higher genetic variability compared to the cultivated olive and some wild trees were found adapted to particularly harsh conditions; therefore, the role of oleaster in the future of olive cultivation may be crucial. Despite the great potential, only recently the need to deeply characterize and adequately preserve the wild olive resources drew the attention of researchers. In this review, we summarized the most important morphological and genetic studies performed on oleaster trees collected in different countries of the Mediterranean Basin. Moreover, we reviewed the strategies introduced so far to preserve and manage the oleaster germplasm collections, giving a future perspective on their role in facing the future agricultural challenges posed by climatic changes and new emerging diseases.

## 1. Introduction

Olive (*Olea europaea* L.) is one of the most iconic trees of Mediterranean Basin, with important implications from a social, economic, and ecological point of view [1]. Six subspecies are currently recognized in the olive species based on morphological traits and geographical distribution [2]. The subsp. *cuspidata* is widely spread in Africa and Asia, subsp. *laperrinei* is present in the Sahara Desert, subsp. *maroccana* occurs mostly in Morocco, and subsp. *guanchica* and *cerasiformis* were found in the Canary Islands and Madeira, respectively [3]. Finally, subsp. *europaea* is uniformly distributed across the whole Mediterranean Basin and is the only subspecies divided into two botanical varieties: cultivated olive (var. *europaea*) and wild olive or oleaster (var. *sylvestris*) [2]. Most of the subspecies are diploid (2n = 46), while subsp. *cerasiformis* is a tetraploid and subsp. *maroccana* has a hexaploid cytotype [3].

Cultivated olive originated from oleaster in northeastern Levant of Mediterranean Basin approximately 6000 years ago [4]. The presence of independent domestication events or a primary center of domestication followed by multiple secondary events is still debated [4,5]. The domestication and selection processes have led to a more productive and highly adaptable tree [4], resulting in a constantly increasing number of varieties. It is estimated that more than 2000 cultivars are grown worldwide [6]; however, the International Olive Council (IOC) estimates that only 139 olive cultivars, cultivated in 23 different countries, account for almost 85% of the world’s olive production [7].

Wild and cultivated olive have overlapping distribution through the Mediterranean Basin even though the presence of oleaster is predominant in the central-western part of the Mediterranean area, where some old-growth forests were found [8]. Cultivated olive and oleaster are distinguishable based on both morphological and genetic differences [9]. Usually, wild olives are shrubs rather than trees, the branches are often spiny, and the fruits are smaller than those of cultivated olives and yield much less oil. Wild olive can be considered as a mixture of micro-varieties that often have characters parallel to those of cultivars, except for the smaller size and reduced oil content of the fruit [10]. 

Beside genuine wild olives, which correspond to natural populations, there are the feral forms, secondary sexual derivatives originated from hybridization between cultivated trees and oleasters, as the two varieties are fully inter-fertile [9]. Feral olive forms were placed in intermediate position based on the morphological traits of tree, leaf, fruit, and stone. Although they are not easily morphologically distinguishable, some studies based on genetic analysis allowed the distinction between feral germplasm and genuine wild olives evidencing the persistence of genuine oleaster populations in several countries of the Mediterranean area [11,12,13,14].

Olive has adapted to grow in diverse climatic conditions with variable altitudes and different soils. Moreover, it can tolerate drought stress and different temperature regimes [6]. Nevertheless, it is not to be excluded that drastic climate changes may affect olive cultivations. A substantial warming and a significant decrease in precipitation are expected to affect the Mediterranean Basin in the next decades [15], leading to serious economic and ecological consequences. Indeed, the climate changes are expected to induce a significant reduction in the olive-suitable growing area and productivity mostly due to the strong adaptation of widely cultivated varieties to specific climatic conditions [6]. Moreover, emerging olive diseases, such as quick decline syndrome (*Xylella fastidiosa* subsp. *pauca*) and olive decline caused by *Pleurostomophora richardsiae*, are generating significant concerns from an economic and ecological point of view [16,17]. Quick decline syndrome caused by *Xylella fastidiosa* is of particular concern, as it has spread quickly in the last eight years, killing millions of olive trees, and is still expanding [17]. Currently, the realization of studies involving a wide number of diverse olive trees aiming to identify sources of resistance is in progress.

In this scenario, the availability of wild olive germplasm resources suitable for breeding programs and the preservation of this great source of genetic diversity is crucially important. The potential of wild olive as a source of resistance was widely proved with *Verticillium dahliae* [18,19] and other biotic and abiotic stresses [20,21,22]. Moreover, the importance of the availability of a large genetic diverse germplasm collection in performing screening aiming to identify resistant genotypes was widely demonstrated [23,24,25,26].

One major concern about the use of wild olive in breeding programs is related to the supposed lower quality, in terms of fatty acids composition and phenolic compounds, of oil compared to extra virgin olive oil (EVOO) obtained from cultivated varieties. However, recent studies demonstrated that the use of wild olive in oil production does not have a negative impact on fatty acid composition, tocopherol, and phenolic composition, suggesting the suitability of oleaster in high-quality oil production [27,28].

In this review, we gave an outline of the most important morphological and genetic studies performed on different wild olive germplasm collections summarizing their principal findings and conclusions. Moreover, we performed a meta-analysis with the purpose of comparing the genetic differentiation found in different oleaster populations and their country of origin. Finally, we focused on the strategies introduced so far to preserve and manage the oleaster collections, giving a future perspective on how the wild olive genetic resources would be important to face the future agricultural challenges posed by climatic changes and new emerging diseases.

## 2. Morphological and Genetic Characterization of Oleaster

Over the last 20 years, several studies focusing on the morphological and genetic characterization of oleaster populations were performed. The aims of these works were (1) to study the origin and domestication of cultivated olive; (2) to distinguish between genuine plants and feral forms; (3) to search out new sources of genetic variability for breeding programs; (4) to investigate the effects of human migration on olive domestication and selection; and (5) to describe the gene flow patterns existing in genuine wild populations. Table 1 summarizes the principal works performed, in order to morphologically and genetically characterize wild olive collections. 

### 2.1. Morphological Evaluation

Morphological evaluation is the basis for olive characterization, but its dependence on plant development stages, the subjectivity of analysis, and the influence of environmental factors on its assessment have led to the progressive use of morphological descriptors in combination with molecular marker-based methods, which are fundamental in the genetic diversity assessment of large populations [58]. 

Most morphological studies aimed to characterize oleaster with the purpose of exploring the diversity existing in isolated populations for the selection of individuals to include in specific conservation programs [45,51,55] and make a comparison with cultivated olive [38,39,44,52,57]. The main analyzed traits were related to the drupe and pit morphology, as well as the oil content and composition. In general, oleaster demonstrated a higher level of variability compared to cultivated olive [24,39,44] with smaller drupe and pit and a lower pulp percentage compared to var. *europaea* [38,39,52]. The oil content and the fatty acid composition demonstrated the same range of variation of cultivated olive, demonstrating the suitability of oleaster in the production of high-quality oil [39,52]. Recently, Khouatmiani et al. [57] assessed the effect of geo-climatic conditions on pollen traits of eight Algerian oleaster populations, founding a correlation between latitude, longitude, and temperature of the site of collection and shape and size of the pollen. Although there are some limitations, morphological descriptors also remain important today for a first olive identification thanks to modern technologies, which allow us to morphologically characterize olive plants through reliable and low-cost automated platforms that significantly reduce the influence of human observation [59,60].

### 2.2. Genetic Diversity Assessment

**Figure 1:**
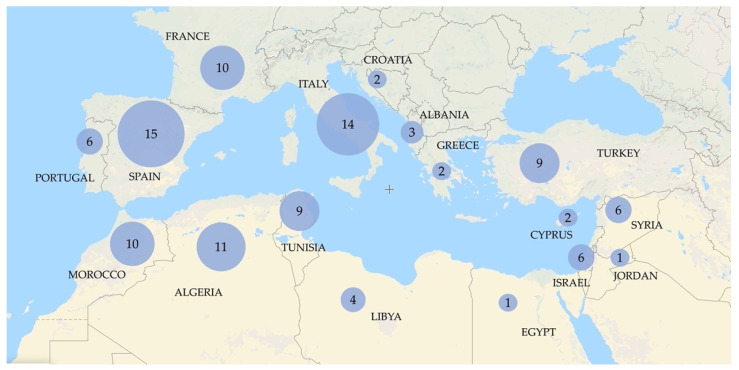
Map showing the countries in which the oleaster trees (*Olea europaea* L. subsp. *europaea* var. *sylvestris)* used in the studies cited in this review were collected. The number of studies performed in each country is indicated.

The first genetic studies involving the oleaster were performed in the early 2000s. The main purposes of these works were the analysis of the history of olive domestication and the investigation of the genetic relationships existing between cultivated olive and genuine oleaster, and between the subsp. *europaea* and other subspecies. For this reason, they included numerous samples collected in different countries of the Mediterranean Basin (Figure 1) [11,30,31,32,33]. In addition, some works also focused on the genetic relationship between cultivated and wild olive in restricted peculiar geographical regions [12,29,34,43].

In 2000, Besnard and Bervillé [11] performed an extensive study based on the use of RFLP (restriction fragment length polymorphism) and RAPD (random amplified polymorphic DNA) markers to analyze about 500 samples, including cultivated olives, oleasters, and trees, belonging to subsp. *maroccana*, *laperrinei*, *cerasiformis,* and *cuspidata*. The authors highlighted the complexity of the genetic relationship between cultivated olive and oleaster, mainly due to the human displacement of cultivars over the centuries. Thereafter, other studies performed with the same molecular markers and ISSR (inter-simple sequence repeat) have come to the same conclusions [31,32].

With the spreading of the use of SSR (simple sequence repeat) or microsatellite markers in olive genetic analysis, the number of studies aiming to dissect the molecular diversity existing in large natural populations of oleaster significantly increased. Most of them focused on large populations of oleaster located in specific areas of the Mediterranean Basin, with the main purposes of studying the genetic diversity within and among wild populations [36,47], investigating the genetic relationships among wild and cultivated olive in a specific geographical area [5,13,40,41,42,48,50,56], setting up an effective program for germplasm conservation [37], and identifying traits of interest for the improvements of cultivated olive [14,24].

The first large-scale molecular study of oleaster based on SSR markers was performed by Breton et al. [35] in 2006 on samples collected in different countries of the Mediterranean Basin. The authors demonstrated that gene flow has occurred in oleasters mediated by cultivars spread by human migration and trade and that native oleasters are still present not only in the eastern Mediterranean but also in the Western side of the Basin, in contrast with previous observations [61]. Belaj et al. [36] confirmed the hybridization between native oleasters and cultivated varieties in areas of close contact between the two forms, as indicated by a high degree of admixture among cultivated and wild populations, pointing out the difficulty to identify clear boundaries between candidate areas containing either genuinely wild or feral germplasm.

In 2013, Besnard et al. [46] performed an extensive study aiming to describe patterns of genetic differentiation in the Mediterranean and Saharan olives through the analysis of more than 1000 olive trees, including cultivated olives, oleasters, and Saharan trees (*O. europaea* subsp. *laperrinei*) by microsatellite markers. The work confirmed the higher genetic diversity in oleaster compared with cultivars, as previously described [39,40,44], while the admixture observed between Mediterranean and Saharan olives led the authors to suppose a role for Laperrine’s olive in the diversification of cultivated olives.

Diez et al. [5] obtained evidence for multiple domestication events and historical admixture between cultivated and wild populations through the analysis of two collections of oleaster and cultivated olive. The results supported the presence of a second and separate olive domestication event in the central Mediterranean Basin.

A large collection of oleaster samples from Spain was genetically analyzed by Beghè et al. [47] in 2017. The main aim of this study was the quantification of the gene flow extent in wild olive natural populations through the quantification of the pollen immigration rate and within-population pollen dispersal distances. The results were demonstrated to be useful in defining programs for the conservation of olive tree forest genetic resources and limiting the effect of anthropogenic activities. Moreover, self-incompatibility and preferential mating between some genotypes were revealed. The work also highlighted the potential represented by the gene pool of wild olive as sources of genetic diversity linked to interesting agronomical and ecophysiological traits. The same concept was also taken into account by Falek et al. [14]. In this study, a large number of wild olives was collected in Algerian sites characterized by various climatic and soil conditions with the purpose of identifying accessions adapted to particularly harsh conditions. The accessions clustered according to their ecogeographic origin, allowing the identification of a group of samples collected from an area characterized by high temperatures and low precipitation. These samples could represent a precious source of genes for tolerance to dry climatic conditions, making them an important resource for future breeding programs. 

Besides the use of nuclear molecular markers, cytoplasmic DNA polymorphism has been used, in particular, to investigate the phylogenetic relationships existing among subspecies and species of genus Olea. Indeed, the nucleotide variability of chloroplast and mitochondrial markers makes them extremely useful for evolutionary purposes. Moreover, cytoplasmic markers are extremely useful in the presence of polyploid species (e.g., subsp. *cerasiformis* and *maroccana*), making the comparison with diploid individuals a more straightforward process. Cytoplasmic markers allowed for the comparison of olive cultivar displacement in the Mediterranean area with the distribution of cytotypes in oleasters collected in different countries [11]; the assessment of the maternal phylogenetic relationships within the Oleaceae family [26,30,46]; and the study of the gene flow via seed movement in a large collection of oleasters [35].

Amane et al. [29], analyzing chloroplast RFLP variation in wild and cultivated olives in Morocco, observed that the chlorotype predominant in the wild and cultivated olive of the whole Mediterranean Basin was observed also in the Moroccan olive germplasm, confirming that cultivated and wild olive material are closely related maternally. Mitochondrial DNA markers proved useful to differentiate between feral and genuine oleasters in Corsica [12]. Chloroplast SSR markers were used by Hannachi et al. [42] to study the variability existing among wild and cultivated olives of Tunisia, demonstrating a diverse origin for Tunisian olive, and confirming strong relationships with autochthonous and introduced cultivars. 

The olive chloroplast DNA sequencing allowed for the development of new chloroplast molecular markers, which was useful to observe a geographic pattern of genetic differentiation that reflects the primary origins of cultivars in the Levant and highlighted a high genetic differentiation between *europaea* and the Saharan olive *laperrinei* [46,62]. More recently, Niu et al. [63] performed an evolutionary analysis by the sequencing of the complete chloroplast genomes of three *O. europaea* subspecies (subsp. *cuspidata*, subsp. *europaea* var. *sylvestris*, and subsp. *europaea* var. Frantoio). The authors showed a high similarity of the chloroplast genome between var. *sylvestris* and var. *europaea*, demonstrating that a few differentiation events were present in the chloroplast DNA of subsp. *europaea*.

The whole genome sequencing of olive cultivar Farga in 2016 [64], oleaster in 2017 [65], and, more recently, cultivar Arbequina [66], gave a boost to the study of genetic diversity existing in Olea species, based on the high-reproducible and effective SNP (single nuclear polymorphism) markers. Large sets of these markers were developed especially for the study of cultivated olive [67,68,69,70]; however, their use has been also extended to the analysis of wild germplasm [49,53,54,71]. Gros Balthazar et al. [53] compared cultivated and wild olives by using transcriptomics, supporting a major domestication event in the eastern part of the Mediterranean basin followed by dispersion towards the West and subsequent admixture with western wild olives. Kyriakopoulou and Kalogianni [72] developed a new technique able to distinguish between wild and cultivated olive based on the analysis of a single SNP through allele-specific, real-time Polymerase Chain Reaction (PCR). The proposed method was demonstrated to be suitable for oil traceability purposes, as it was able to detect as little as 1% content of the oleaster in binary DNA mixtures of the two olive species. 

Using the whole genome resequencing of twelve olive samples, including one oleaster, Julca et al. [73] studied the recent evolution and domestication of the olive tree, confirming the olive primary domestication in the eastern Mediterranean basin followed by numerous secondary events across different countries, often involving genetic admixture with genetically rich wild populations, particularly from the western Mediterranean Basin.

From all the studies about oleaster carried out so far, some key concepts have emerged and they are summarized in Table 2. The evaluation and characterization of local wild germplasm populations performed in these studies pointed out the great importance and relevance of these collections, as they represent a great source of genetic variability [14,47,74].

### 2.3. Correlation between the Oleaster Population Genetic Differentiation and the Country of Origin

Genetic studies about oleaster populations tried to dissect the variability existing within and between populations and described their genetic structure. Among the different genetic diversity indices, the fixation index (F_ST_) is one of the most used. The F_ST_ was introduced by Wright [75], and it measures the genetic difference between subpopulations. Different authors tried to estimate the fixation index; the most commonly used estimator is the one proposed by Nei [76], who correlates the F_ST_ with expected and observed heterozygosity. A positive F_ST_ value indicates lower heterozygosity than expected due to inbreeding; on the contrary, a negative value means an excess of heterozygotes. A negative F_ST_ value may be due to the presence of factors contributing to increasing heterozygosity, such as the crossing with individuals belonging to other populations and a large number of widely dispersed individuals composing the population.

With the aim to compare the genetic differentiation existing inside oleaster populations and the country of origin, we performed a meta-analysis of different studies to investigate the average F_ST_ in oleaster populations originating in different countries of the Mediterranean Basin. The criterion for including published studies in our meta-analysis was that they reported the mean F_ST_ values or, alternatively, mean observed and expected heterozygosity values, the population sizes, and the origin of the samples. The final data set consisted of 13 articles (Supplementary Materials Table S1).

**Figure 2:**
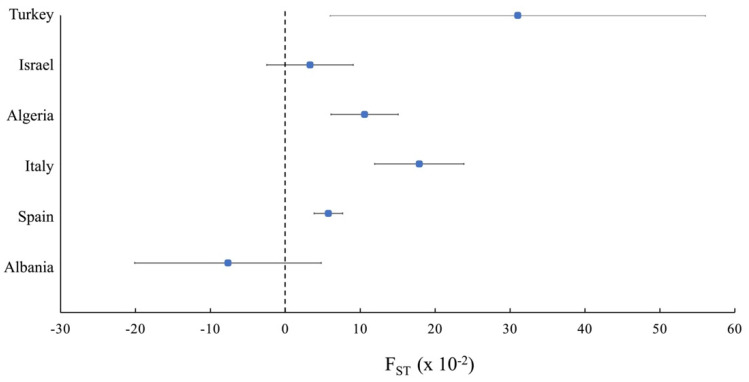
Forest plot showing the comparison of the mean fixation index F_ST_ in populations collected in different countries of the Mediterranean Basin.

The meta-analysis was performed following Neyeloff et al. [77]. The outcome is represented by the mean fixation index F_ST_ value obtained for each population. When more than one population was analyzed in the same country, the weighted average F_ST_ value was used. Figure 2 shows the results obtained from the meta-analysis. The mean F_ST_ assumes values between −0.08 and 0.31. For all the analyzed countries, except Albania, F_ST_ presents a positive value, indicating some degree of inbreeding into the oleaster populations. Although olive presents different mechanisms to promote outbreeding, such as self-incompatibility [78], inbreeding is quite spread in oleaster, especially in populations geographically isolated [14,35,41], inducing a severe reduction of genetic variability [79]. This can lead to dramatic consequences due to inbreeding depression [80].

In the perspective of the climatic changes and the occurrence of new olive diseases, the availability of wild olive trees highly adapted to harsh environments becomes of crucial importance. Thus, the adoption of appropriate conservation strategies and the efficient management of oleaster genetic resources are necessary to properly preserve these important resources of traits of interest, in order to efficiently use them in targeted breeding programs and safeguard their genetic variability that may be seriously reduced, especially in the highly isolated populations. 

## 3. Conservation of Oleaster Genetic Resources 

Crop wild relatives represent a high value due to their greater genetic variation compared to crops and constitute a precious reservoir of traits useful for developing more productive, high-quality, and resilient crop varieties. The importance of adopting an appropriate and effective conservation strategy for wild relatives was widely demonstrated [81,82]. In the last years, particular attention was given to the characterization and evaluation of local and peculiar varieties, and targeted conservation programs have been established [83,84,85,86,87,88]. On the contrary, the efforts made to preserve and adequately conserve the wild germplasm collections were limited and fragmented.

All the studies about genetic and morphological evaluation of wild olive populations demonstrated an outstanding variability, most of which was not present in the cultivars. However, for a long time, wild olives have been considered of low agronomical value and they have only occasionally been used in olive breeding programs, which were mostly based on intra-specific crosses between cultivars [89]. Only in the last few years has the identification of oleasters growing in arid regions at different altitudes and soil types, adapted to different adverse environmental conditions [13,14,34], along with the acknowledgment of the value of extra virgin olive oil produced with wild trees [28,29], drawn the attention on the necessity to conserve and adequately manage oleaster genetic resources. The preservation and protection of the wild olive also assumes a great relevance, following the increasing loss of large old oleaster forests due to the deforestation that is becoming considerably extensive in some Mediterranean areas [8].

In general, olive accessions have been maintained in ex situ field collections, which permit an easy management of accessions, reduce the effects of biotic and abiotic stresses, minimize the loss of total variation, and ensure optimal use of genetic resources in breeding programs. However, the in situ conservation allows the coevolution of genotypes in their original environment and the conservation of ancient trees. Therefore, the implementation of a conservation program based on both in situ and ex situ approaches represents the most complete and successful strategy for the optimal management and use of olive genetic resources.

Diverse olive genebanks are available worldwide, most of them containing several international varieties and local cultivars. Oleaster accessions are present only in the largest international genebanks and in a few national in situ and ex situ germplasm collections (Table 3). 

In 1994, the International Olive Council (IOC) created an international network of germplasm banks as part of the RESGEN project. This network is composed of three international banks located in Córdoba (Spain), Marrakech (Morocco), and Izmir (Turkey), and 20 national banks (Albania, Algeria, Argentina, Croatia, Cyprus, Egypt, France, Greece, Iran, Israel, Italy, Jordan, Lebanon, Libya, Montenegro, State of Palestine, Portugal, Slovenia, Tunisia, and Uruguay) [7]. 

The Worldwide Olive Germplasm Bank of Córdoba in Spain (WOGC—IFAPA) was established in 1970 and represented the first international attempt of the conservation and management of the olive germplasm. This is one of the largest olive germplasm collections, encompassing about 500 accessions from 21 countries [90]. It includes an extensive ex situ collection of oleaster germplasm from several sites, representing adverse and heterogeneous ecological conditions. This wild olive collection was evaluated with morphological and genetic markers [40,44,90]. In addition, a wild albino ivory-white olive tree was identified in Medes Island, a protected natural reserve in the Northwest Mediterranean Sea. The germplasm was surveyed from the IFAPA center of Cordoba and preserved ex situ at the Institute of Agrifood Research and Technology (IRTA, Constantí, Spain) [91].

A second Worldwide Olive Germplasm Bank was created at the INRA Research Station of Tassaoute, Marrakech (Morocco, WOGBM) in 2003. This worldwide collection includes almost 560 accessions collected from 14 Mediterranean countries and it was genetically and morphologically characterized [90,92]. In 2012, a third worldwide germplasm bank was established at the Experimental Station of the Olive Research Institute in Izmir (Turkey), including about 300 accessions from different Mediterranean countries [93]. In 2015, a collaboration between the three collections of Marrakech, Córdoba, and Izmir was established for promoting the exchange of plant material between the three collections.

At a national level, an example of wild germplasm conservation is represented by the Gene Bank of the Agricultural University of Tirana, Albania, which preserves several accessions belonging to var. *sylvestris* collected throughout the country, from farms, in situ, and ex situ collections [51]. Some of the oleaster trees were evaluated through morphological and SSR markers [45,51,56]. Another national collection including several wild olives is the National Olive Germplasm Bank present in Izmir (Turkey), beside the international collection [93]. In addition, the National Olive Genebank of Tunisia deserves a mention. Here, the sustainable management and preservation of genetic resources are performed through ex situ and on farm conservation strategies. The collection contains several accessions, including the wild relatives of cultivated plants [94].

Despite the efforts to include oleaster trees in these in situ and ex situ collections, great work is still needed to protect and preserve the huge genetic resource represented by the wild olive germplasm. The efforts made by the IOC, which has established a common guide to authenticate, sanitize, preserve and exchange plant material between banks, are essential to connect the ministries and the national supply agencies with the scientific communities. It is hoped that among the future goals of the IOC network there will also be greater attention to the identification and collection of wild olive trees through specific actions aimed at preserving them through appropriate conservation strategies.

## 4. Conclusions and Future Perspective 

Recently, we have faced rapid climate change in most of the world’s regions; the Mediterranean Basin represents one of the most affected areas. Furthermore, these significant modifications are expected to progress in the next decades, severely affecting the cultivations of several crops, including olive [6,15]. In addition, new diseases have emerged in the few last years, causing severe damages to the olive groves and the loss of some ancient monumental trees [16,17,95]. Therefore, the introduction of measures aiming to face new agricultural challenges and avoid the loss of the precious Mediterranean olive heritage is fundamental.

Oleaster constitutes a valuable source of genetic variability with huge potential for olive breeding, also thanks to the inter-fertile nature of the *europaea* and *sylvestris* varieties. Moreover, it is notable that oleasters are spread all over the Mediterranean Basin and demonstrate an overlapping distribution with the cultivated olive [8], making them an easily accessible source of traits of interest. Alternatively, the wild accessions can be used as rootstocks, bringing important benefits to the tree. This approach was widely employed in ancient cultivation systems, but it seems to be much less used in modern olive crops [96]. However, the important improvements demonstrated by grafted plants, such as the enhanced productivity of high-density hedgerow orchards [24], makes possible the return to the use of oleaster as rootstock for some cultivars.

In the last years, the number of studies aiming to dissect the morphological and genetic variability existing in the populations of oleasters dispersed through the different Mediterranean countries has progressively grown. However, better knowledge and exploitation of wild olive is still needed. A thorough phenotyping, an accurate agronomical evaluation, and the application of high-throughput genotyping are fundamental to obtain an exhaustive comprehension of the variability existing in wild populations and identify the agronomic traits useful for future breeding programs. 

Extensive knowledge of large wild tree collection is also required to properly conserve and manage these resources in order to efficiently plan the exploration and collection of accessions, ensuring that the entire gene pool is adequately represented and minimizing redundancy. Several programs have been established all over the world aiming to conserve olive trees, but only a few of them included oleaster accessions. Some efforts have been made at the regional level; however, global and coordinated action is desirable to properly preserve the wealthy inheritance represented by these trees. The ex situ conservation is the easiest way to manage and protect olive genetic resources, although their conservation in their natural habitats is essential to maintain the genetic diversity existing in a wild population, which is continually adapting to local environmental conditions. The best approach would be to apply a combination of in situ and ex situ conservations, allowing the advantages of each method to complement each other. A single research center cannot efficiently conserve and preserve the numerous oleaster collections present in a specific territory; therefore, a wide international network is required to evaluate and catalog all the genetic resources of a certain area, ensuring the adoption of the best conservation strategy and management approach.

For a long time, oleaster has been considered of lower agronomical value compared to the cultivated olive; however, the growing interest of researchers in its morphological, genetic, and chemical characterization has highlighted the huge genetic variability existing in populations of wild trees, their adaptability to harsh climatic conditions, such as dry and warm climates, and their suitability in the production of high-quality extra virgin olive oil, making these resources extremely precious when facing the challenges posed by climatic changes and new emerging olive diseases. The awareness of the great value represented by oleaster will boost the intention to valorize and protect this important and precious resource for the future of the olive species.

## Figures and Tables

**Table 1:** List of the most important morphological and genetic studies performed on wild olive. The analyzed subspecies and varieties of *O. europaea*, the number of wild accessions, and the used markers are indicated.

Reference	Analyzed Subspecies	Analyzed Varieties of Subspecies *europaea*	Number of Analyzed Oleaster Accessions	Used Markers
Amane et al., 2000 [29]	*europaea, laperrinei*	*europaea* and *sylvestris*	45	RFLP
Besnard and Bervillé, 2000 [11]	*europaea, maroccana, laperrinei, cerasiformis, cuspidata*	*europaea* and *sylvestris*	300	RAPD, RFLP
Lumaret et al., 2000 [30]	*europaea, maroccana, laperrinei, cuspidata*	*europaea* and *sylvestris*	101	RFLP
Besnard et al., 2001 [31]	*europaea, maroccana, laperrinei, cerasiformis, cuspidata*	*europaea* and *sylvestris*	292	RAPD, RFLP
Vargas and Kadereit, 2001 [32]	*europaea, maroccana, laperrinei, cerasiformis, cuspidata, guanchica*	*europaea* and *sylvestris*	26	ISSR
Besnard and Bervillé, 2002 [33]	*europaea, maroccana, laperrinei, cerasiformis, cuspidata*	*europaea* and *sylvestris*	7	RFLP
Bronzini de Caraffa et al., 2002 [12]	*europaea*	*europaea* and *sylvestris*	99	RAPD, RFLP
Baldoni et al., 2006 [34]	*europaea*	*europaea* and *sylvestris*	100	AFLP
Breton et al., 2006 [35]	*europaea*	*sylvestris*	166	SSR
Belaj et al., 2007 [36]	*europaea*	*sylvestris*	171	SSR
Brito et al., 2008 [37]	*europaea, cerasiformis, guanchica*	*europaea* and *sylvestris*	8	SSR
Hannachi et al., 2008 [38]	*europaea*	*europaea* and *sylvestris*	70	Morphological, SSR
Hannachi et al., 2009 [39]	*europaea, cuspidata*	*europaea* and *sylvestris*	12	Morphological, SSR
Belaj et al., 2010 [40]	*europaea*	*europaea* and *sylvestris*	107	SSR
Erre et al., 2010 [41]	*europaea*	*europaea* and *sylvestris*	21	SSR
Hannachi et al., 2010 [42]	*europaea*	*europaea* and *sylvestris*	52	SSR
Sesli and Yegenoglu, 2010 [43]	*europaea*	*europaea* and *sylvestris*	8	RAPD
Belaj et al., 2011 [44]	*europaea*	*sylvestris*	48	Morphological, SSR
Ismaili et al., 2012 [45]	*europaea*	*sylvestris*	27	Morphological
Besnard et al., 2013 [46]	*europaea, laperrinei*	*europaea* and *sylvestris*	390	SSR
Diez et al., 2015 [5]	*europaea*	*europaea* and *sylvestris*	96	SSR
Beghè et al., 2017 [47]	*europaea*	*sylvestris*	225	SSR
Boucheffa et al., 2017 [48]	*europaea*	*europaea* and *sylvestris*	16	SSR
Chiappetta et al., 2017 [13]	*europaea*	*europaea* and *sylvestris*	99	SSR
Belaj et al., 2018 [49]	*europaea*	*europaea* and *sylvestris*	89	SNP
di Rienzo et al., 2018 [50]	*europaea*	*europaea* and *sylvestris*	16	SSR
Ismaili et al., 2018 [51]	*europaea*	*europaea* and *sylvestris*	61	Morphological, SSR
Boucheffa et al., 2019 [52]	*europaea*	*europaea* and *sylvestris*	12	Morphological, SSR
Gros-Balthazard et al., 2019 [53]	*europaea, cuspidata*	*europaea* and *sylvestris*	27	SNP
Díaz-Rueda et al., 2020 [24]	*europaea, maroccana, laperrinei, cerasiformis, cuspidata, guanchica*	*europaea* and *sylvestris*	59	Morphological, SSR
Mariotti et al., 2020 [54]	*europaea, guanchica*	*europaea* and *sylvestris*	73	SNP
Rodrigues et al., 2020 [55]	*europaea*	*sylvestris*	12	Morphological
Dervishi et al., 2021 [56]	*europaea*	*europaea* and *sylvestris*	19	SSR
Khouatmiani et al., 2021 [57]	*europaea*	*sylvestris*	24	Morphological
Falek et al., 2022 [14]	*europaea*	*sylvestris*	174	SSR

RFLP (restriction fragment length polymorphism); RAPD (random amplified pol-ymorphic DNA); ISSR (inter-simple sequence repeat); SNP (single nuclear polymorphism); AFLP (Amplified fragment length polymorphism).

**Table 2:** Summary of the principal findings of the morphological and genetic studies performed on oleaster so far. The corresponding references are also indicated.

Key Findings	Reference
Multiple domestication events took place in olive	Diez et al., 2015 [5] Gros-Balthazard et al., 2019 [53] Julca et al., 2020 [73]
Wild olive includes feral forms and genuine wild olives	Bronzini de Caraffa et al., 2002 [12] Baldoni et al., 2006 [34] Breton et al., 2006 [35] Chiappetta et al., 2017 [13]
Genuine oleasters show a much higher level of morphological and genetic variability compared to cultivated olives	Hannachi et al., 2009 [39] Belaj et al., 2010 [40] Belaj et al., 2011 [44] Besnard et al., 2013 [46] Díaz-Rueda et al., 2020 [24]
A constant gene flow takes place in the regions in which wild and cultivated olives coexist, making the distinction difficult between genuinely wild and feral olive	Besnard et al., 2001 [31] Bronzini de Caraffa et al., 2002 [12] Breton et al., 2006 [35] Belaj et al., 2007 [36] Boucheffa et al., 2017 [48]
Wild olive is an important source of traits related to biotic and abiotic stress tolerances.	Beghè et al., 2017 [47] Mariotti et al., 2020 [54] Falek et al., 2022 [14]

**Table 3:** List of germplasm collections, including oleaster accessions.

Institution	Country	Strategy of Conservation
WOGC—IFAPA, Worldwide Olive Germplasm Bank of Córdoba	Spain	Ex situ
WOGB—INRA, Worldwide Olive Germplasm Bank of Marrakech	Morocco	Ex situ
WOGB—Worldwide Olive Germplasm Bank of Izmir	Turkey	Ex situ
National Gene Bank of Tunisia (NGBT)	Tunisia	Ex situ and in situ
Olive Gene Bank of Albania	Albania	Ex situ and in situ
National Olive Germplasm Bank of Turkey	Turkey	Ex situ and in situ

## Data Availability

Not applicable.

## Supplementary Materials

The following supporting information can be downloaded at: https://www.mdpi.com/article/10.3390/plants11040480/s1, Table S1: list of studies selected for the meta-analysis.
