# Supplementary material for: Current Status of Biodiversity Assessment and Conservation of Wild Olive (Olea europaea L. subsp. europaea var. sylvestris)
Source: Plants (Basel). 2022 Feb 10;11(4):480. doi: 10.3390/plants11040480 (PMC8877956; doi:10.3390/plants11040480)
Supplement: Supplementary file 1 [file plants-11-00480-s001.zip › plants-1569289 R2 supplementary.pdf]

plants

Review

Current status of biodiversity assessment and conservation of wild olive (*Olea europaea* L. subsp. *europaea* var. *sylvestris*)

Valentina Fanelli [1,*], Isabella Mascio [1], Wahiba Falek [2], Monica Marilena Miazzi [1,*] and Cinzia Montemurro [1,3,4]

1 Department of Soil, Plant and Food Sciences, University of Bari Aldo Moro, via Amendola 165/A, Bari, 70125, Italy; mascioisa@gmail.com (I.M.); cinzia.montemurro@uniba.it (C.M.)
2 Ecole Nationale Supérieure de Biotechnologie, Ville universitaire Ali Mendjeli, BP E66 – 251000 – Constantine, Algeria; wfalek@ymail.com (W.F.)
3 Spin off Sinagri s.r.l., University of Bari Aldo Moro, via Amendola 165/A, Bari, 70125, Italy
4 Institute for Sustainable Plant Protection–Support Unit Bari, National Research Council of Italy (CNR), Via Amendola 165/A, Bari, 70125, Italy
* Correspondence: valentina.fanelli@uniba.it (V.F.); monicamarilena.miazzi@uniba.it (M.M.M.)

Supplementary Material

Table S1. Population size and country of origin of samples analyzed in the studies included in the meta-analysis.

Reference	Country of origin	Population size
Belaj et al., 2007	Spain	107
	Italy	64
Belaj et al., 2010	Spain	107
Erre et al., 2010	Italy	21
Belaj et al., 2011	Spain	48
Diez et al., 2015	Israel	38
	Spain	48
	Italy	10
Beghè et al., 2017	Spain	225
Boucheffa et al., 2017	Algeria	16
Chiappetta et al., 2017	Italy	99
Boucheffa et al., 2019	Algeria	12
Gros-Balthazard, 2019	Turkey	19
Mariotti et al., 2020	Spain	73
Dervishi et al., 2021	Albania	19
Falek et al., 2022	Algeria	174
